# Foam-like phantoms for comparing tomography algorithms

**DOI:** 10.1107/S1600577521011322

**Published:** 2022-01-01

**Authors:** Daniël M. Pelt, Allard A. Hendriksen, Kees Joost Batenburg

**Affiliations:** a LIACS, Leiden University, Leiden, The Netherlands; b Computational Imaging Group, CWI, Amsterdam, The Netherlands

**Keywords:** tomography, phantom, simulation, open-source, experiment design

## Abstract

Tomographic algorithms are often compared by evaluating them on certain benchmark datasets. For fair comparison, these datasets should ideally (i) be challenging to reconstruct, (ii) be representative of typical tomographic experiments, (iii) be flexible to allow for different acquisition modes, and (iv) include enough samples to allow for comparison of data-driven algorithms. Current approaches often satisfy only some of these requirements, but not all. For example, real-world datasets are typically challenging and representative of a category of experimental examples, but are restricted to the acquisition mode that was used in the experiment and are often limited in the number of samples. Mathematical phantoms are often flexible and can sometimes produce enough samples for data-driven approaches, but can be relatively easy to reconstruct and are often not representative of typical scanned objects. In this paper, we present a family of foam-like mathematical phantoms that aims to satisfy all four requirements simultaneously. The phantoms consist of foam-like structures with more than 100000 features, making them challenging to reconstruct and representative of common tomography samples. Because the phantoms are computer-generated, varying acquisition modes and experimental conditions can be simulated. An effectively unlimited number of random variations of the phantoms can be generated, making them suitable for data-driven approaches. We give a formal mathematical definition of the foam-like phantoms, and explain how they can be generated and used in virtual tomographic experiments in a computationally efficient way. In addition, several 4D extensions of the 3D phantoms are given, enabling comparisons of algorithms for dynamic tomography. Finally, example phantoms and tomographic datasets are given, showing that the phantoms can be effectively used to make fair and informative comparisons between tomography algorithms.

## Abstract 2

A family of foam-like mathematical phantoms for comparing tomography algorithms is presented.

## 1. Introduction

In tomographic imaging, an image of the interior of a scanned object is obtained by combining measurements of some form of penetrating wave passing through the object. Tomographic imaging is routinely used in a wide variety of application fields, including medical imaging (Goo & Goo, 2017 ▸), materials science (Salvo *et al.*, 2003 ▸), biomedical research (Metscher, 2009 ▸), and industrial applications (De Chiffre *et al.*, 2014 ▸). To extract relevant information from the acquired data, the measurements are often processed by several mathematical algorithms in a processing pipeline. Common processing steps include tomographic reconstruction (Kak *et al.*, 2002 ▸; Marone & Stampanoni, 2012 ▸; Ravishankar *et al.*, 2020 ▸), artifact removal (Barrett & Keat, 2004 ▸; Münch *et al.*, 2009 ▸; Miqueles *et al.*, 2014 ▸), and image segmentation (Iassonov *et al.*, 2009 ▸; Foster *et al.*, 2014 ▸; Perciano *et al.*, 2017 ▸). Because of the importance of tomography in practice, a wide variety of algorithms have been developed for these processing steps, and tomographic algorithm development remains an active research field. In addition to classical image processing algorithms, the use of *data-driven* machine learning algorithms has become popular in tomography in recent years (Jin *et al.*, 2017 ▸; Yang *et al.*, 2017 ▸; Pelt *et al.*, 2018 ▸; Adler & Öktem, 2018 ▸; Liu *et al.*, 2020 ▸; Yang *et al.*, 2020 ▸).

To properly assess the available algorithms, it is essential to compare them with each other in a fair, reproducible, and representative way. Such comparisons are important for algorithm developers to understand how newly developed algorithms compare with existing approaches. Proper comparisons are also important for end users of tomographic imaging to learn which algorithms to use for certain experimental conditions, and to know what results to expect from each available algorithm. A common approach to compare tomography algorithms is to take a set of tomographic datasets, apply several algorithms to the data, and compare results. To make this approach as informative as possible, the chosen datasets should ideally satisfy the following requirements:

(i) The datasets should be **challenging**: it should not be trivial to obtain accurate results for them.

(ii) The datasets should be **representative** of typical objects, experimental conditions, and data sizes that are used in practice.

(iii) The datasets should be **flexible** with respect to object complexity and experimental properties, making it possible to explore the capabilities and limitations of each algorithm for different acquisition modes, experimental conditions, and object complexities.

(iv) The datasets should include enough samples to allow for the comparison of **data-driven** algorithms that require a large number of similar samples for training and testing.

The datasets that are used to compare algorithms in the current literature typically satisfy some of the requirements above, but not all. For example, real-world datasets from public databases (Hämäläinen *et al.*, 2015 ▸; McCollough *et al.*, 2017 ▸; Jørgensen *et al.*, 2017 ▸; Singh *et al.*, 2018 ▸; De Carlo *et al.*, 2018 ▸; Der Sarkissian *et al.*, 2019 ▸) are often used for comparison. While these datasets are both challenging and representative since they are obtained in actual tomographic experiments, they are typically not flexible as it is impossible to change the acquisition mode and experimental conditions that were used in the experiment. In addition, while some datasets are specifically designed for data-driven applications (McCollough *et al.*, 2017 ▸; Der Sarkissian *et al.*, 2019 ▸), other real-world datasets are often not suitable for data-driven approaches, since the number of scanned samples is often limited.

A common alternative to comparing results for real-world datasets is to use computer-generated phantom images for which virtual tomographic datasets are computed. One advantage of using such mathematical phantoms is that the true object is readily available, allowing one to compute accuracy metrics with respect to an objective ground truth. Another advantage is that this approach is flexible: since the tomographic experiment is performed virtually, different acquisition modes and experimental conditions can be easily simulated.

Popular examples of phantoms used in tomography include the Shepp–Logan head phantom (Shepp & Logan, 1974 ▸), the FORBILD head phantom (Yu *et al.*, 2012 ▸), and the MCAT phantom (Segars & Tsui, 2009 ▸). In addition to predefined phantom images, several software packages have been recently introduced that allow users to design their own custom mathematical phantoms and generate simulated tomography datasets for them (Ching & Gürsoy, 2017 ▸; Faragó *et al.*, 2017 ▸; Kazantsev *et al.*, 2018 ▸). The main disadvantage of popular mathematical phantoms is that they typically consist of a small number of simple geometric shapes (*i.e.* less than 100). As a result, the phantoms are often not representative of real-world objects, which typically contain a much larger number of more complicated features. Several often-used phantoms, *e.g.* the Shepp–Logan head phantom, consist of large uniform regions and can therefore be relatively easy to reconstruct accurately using certain algorithms, making it difficult to compare algorithms using these phantoms. Finally, predefined phantoms usually consist of only a single sample and manually defined phantoms require considerable time to design, making it difficult to effectively use them for data-driven approaches that require multiple samples for training.

Because of the aforementioned disadvantages of both real-world datasets and mathematical phantoms, a hybrid approach is used in practice as well (Adler & Öktem, 2018 ▸; Leuschner *et al.*, 2019 ▸; Hendriksen *et al.*, 2020 ▸). In this approach, reconstructed images of real-world tomographic datasets are treated as phantom images for which virtual tomographic datasets are computed. In this way, different acquisition modes and experimental conditions can be simulated for realistic phantom images. However, the approach has several disadvantages when comparing algorithms with each other. First, the reconstructed images have to be represented on a discretized voxel grid and often include various imaging artifacts, resulting in inaccurate representations of the actual scanned objects. Second, the approach can lead to the ‘inverse crime’, *i.e.* when the same image formation model is used for both data generation and reconstruction, which can lead to incorrect and misleading comparison results (Guerquin-Kern *et al.*, 2012 ▸). Finally, since imaging artifacts such as noise and data sampling artifacts are present in the phantom images, artifact-free objective ground truth images with which to compute accuracy metrics are not readily available.

To summarize, new datasets are needed that satisfy all requirements given above for improved comparisons between tomography algorithms. In this paper, we present a family of mathematically defined phantoms that aim to satisfy all requirements. The phantoms consist of three-dimensional foam-like structures and can include more than 100000 features. Since foam-like objects are often investigated using tomography (Babin *et al.*, 2006 ▸; Roux *et al.*, 2008 ▸; Hangai *et al.*, 2012 ▸; Raufaste *et al.*, 2015 ▸; Evans *et al.*, 2019 ▸), the proposed phantoms are representative of a popular class of objects. Furthermore, foam-like objects are typically challenging to accurately reconstruct and analyze due to the fact that they exhibit both large-scale and fine-scale features (Brun *et al.*, 2010 ▸), making them well suited for comparing tomography algorithms. Tomographic datasets can be computed for the proposed phantoms for a wide variety of experimental conditions and acquisition modes and with data sizes that are common in real-world experiments, making the approach both flexible and representative. Finally, an effectively unlimited number of random variations of samples can be generated, enabling comparisons of data-driven algorithms that require multiple samples for training.

The proposed family of simulated foam phantoms has already been used for comparing algorithms in several papers from various research groups (Pelt *et al.*, 2018 ▸; Hendriksen *et al.*, 2019 ▸, 2020 ▸; Liu *et al.*, 2020 ▸; Etmann *et al.*, 2020 ▸; Marchant *et al.*, 2020 ▸; Renders *et al.*, 2020 ▸; Ganguly *et al.*, 2021 ▸). In this paper, a formal definition of the phantoms is given, and mathematical and computational details about both the phantom generation and tomographic experiment simulation are discussed. This paper is structured as follows. In Section 2.1 a mathematical description of the foam phantoms is given, and in Section 2.2 we introduce an algorithm that can compute such phantoms efficiently. We explain how, given a generated foam phantom, tomographic projections can be computed in Section 2.3. Several 4D (*i.e.* dynamic) variations of the proposed phantoms are introduced in Section 2.4. In Section 3, several experiments are performed to investigate the influence of various parameters on the generated phantoms, the computed projection data, and the final tomographic reconstruction. Furthermore, we discuss the required computation time for generating phantoms and computing projection data. In Section 4, we give a few concluding remarks.

## 2. Method

**Figure 1:**
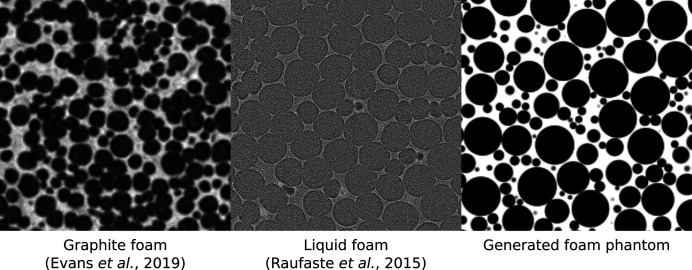
Two examples of X-ray computed tomography images of foam samples (left, middle), and an example of a computer-generated phantom from the proposed foam phantom family (right). In all three images, a cropped region of the entire sample is shown. A graphite foam sample (Evans *et al.*, 2019 ▸; Evans, 2019 ▸) is shown on the left, and a liquid foam sample (Raufaste *et al.*, 2015 ▸), available at Tomobank (De Carlo *et al.*, 2018 ▸), is shown in the middle.

In this section, we give the mathematical definition of the proposed family of phantoms, and describe how such phantoms can be efficiently generated. In addition, we explain how projection images can be computed for both parallel-beam and cone-beam geometries. As explained above, the main inspiration for the design of these phantoms is the continued popularity of investigating a wide variety of real-world foam samples using tomography. In Fig. 1 ▸, two examples are given of such samples, in addition to an example of a foam phantom from the proposed family of phantoms, showing the similarities in features between the proposed foam phantoms and real-world foam samples.

### 2.1. Mathematical description

**Figure 2:**
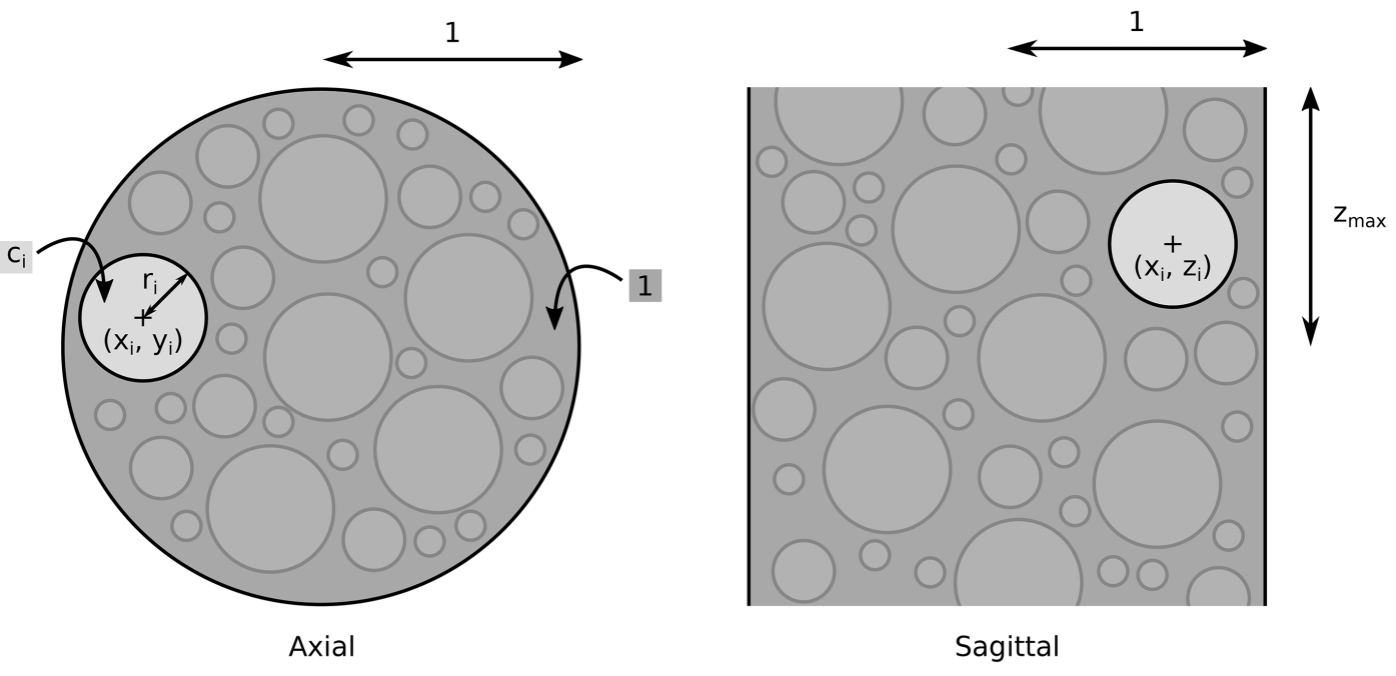
Schematic representation of a foam phantom, with an axial (*i.e.* horizontal) slice shown on the left, and a sagittal (*i.e.* vertical) slice shown on the right. The radius of the main cylinder is fixed to 1, with all other distances defined relative to this radius. Similarly, the attenuation coefficient of the main cylinder is fixed to 1 as well. Each void is characterized by its position *x*_*i*_, *y*_*i*_, and *z*_*i*_, its radius *r*_*i*_, and its attenuation coefficient *c*_*i*_. For one highlighted void, these parameters are shown in the figure. The vertical size of the phantom is defined by *z*_max_. Note that *z*_max_ limits the position of the center of each void, which means that parts of a void can exist at positions larger than *z*_max_ or smaller than −*z*_max_.

In short, each phantom from the foam phantom family consists of a single-material cylinder with a large number (*e.g.* >100000) of non-overlapping spheres of a different material (or multiple different materials) inside. A more detailed explanation follows. Each phantom is defined in continuous 3D space 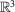 . In all phantoms, a cylinder is placed in the origin, parallel to the *z*-axis (the rotation axis). This cylinder has an infinite height and a radius of 1, with all other distances defined relative to this unit radius. Inside the main cylinder, *N* non-overlapping spheres are placed, which will be called *voids* in the rest of this paper. Each void *i* has a radius 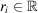 and a position 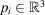 with *p*_*i*_ = (*x*_*i*_, *y*_*i*_, *z*_*i*_). The area outside the main cylinder consists of a background material that does not absorb any radiation, *i.e.* all positions (*x*, *y*, *z*) with (*x* ^2^ + *y* ^2^)^1/2^ > *r*_C_ where *r*_C_ is the radius of the main cylinder (defined to be *r*_C_ = 1). The foam itself, *i.e.* all positions that are within the main cylinder but not inside a void, consists of a single material with an attenuation coefficient of 1, with all other attenuation coefficients defined relative to this. Each separate void in the phantom can be filled with a different material, each with its own user-defined attenuation coefficient 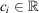 . In the default settings, all voids are filled with the background material. To summarize, each void *i* can be completely characterized by a vector 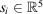 with five elements: its position *x*_*i*_, *y*_*i*_, and *z*_*i*_, its radius *r*_*i*_, and its attenuation coefficient *c*_*i*_. Similarly, each foam phantom is completely characterized by the set of *s*_*i*_ vectors of all voids *S* = {*s*_1_, *s*_2_,…, *s*_*N*_}. The definition of a foam phantom is shown graphically in Fig. 2 ▸.

The vertical size of a phantom is controlled by ensuring that the *z*_*i*_ position of each void *i* satisfies 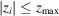 , with a maximum position 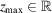 . In addition, no part of any void is allowed to exist outside the main cylinder, *i.e.* 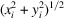 + 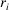 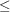 1 for all voids. Also, no part of any void is allowed to overlap with any other void, *i.e.* *d*(*p*_*i*_, *p*_*j*_) ≥ *r*_*i*_ + *r*_*j*_ for all voids *i* and *j*, where *d*(*a*, *b*) is the Euclidian distance between points *a* and *b*. Finally, the size of the voids is controlled by choosing a maximum radius *r*_max_ and ensuring that *r*_*i*_ ≤ *r*_max_ for all voids.

### 2.2. Phantom generation

Foam phantoms are generated by starting with the main cylinder and repeatedly adding voids until *N* voids are placed. When placing the *i*th void at position *p*_*i*_, the radius of the new void is limited by three considerations: (1) the distance to the outside of the main cylinder, 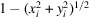 , (2) the distance to the closest edge of any existing void, *i.e.* 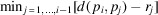 , and (3) the maximum allowed radius *r*_max_. The final radius *r*_*i*_ is chosen to be as large as possible, meaning that

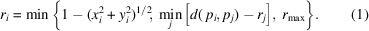

Since placed voids are made as large as possible, the size of newly placed voids naturally becomes smaller during the generation of the phantom: at the end of phantom generation, not much room is left for any new voids, resulting in smaller sizes. Another consequence is that each void *i* either touches the outside of the main cylinder, *i.e.* 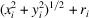 = 1, or touches at least one other void, *i.e.* *d*(*p*_*i*_, *p*_*j*_) = *r*_*i*_ + *r*_*j*_ for some *j*. As a result, the radius of none of the voids can be increased without either making the void overlap another void or having part of the void outside the main cylinder.^1^

We found empirically that realistic looking phantoms (see Fig. 1 ▸) are obtained when new voids are placed in positions that allow for the largest possible void size out of all possible positions. Finding such optimal positions given a partial set of voids is not trivial, since the number of possible positions is, in theory, infinite. We note that it might be possible to deterministically find an optimal position in a computationally efficient way by using a plane sweep algorithm approach (Nievergelt & Preparata, 1982 ▸). However, we propose to use a much simpler approach: a set of *N*_p_ randomly picked *trial points* is available at all times, in which each trial point is a valid position where a void could be placed (*i.e.* inside the main cylinder but not inside an existing void). Then, out of all trial points, a void is placed at the point that results in the largest void. There are several advantages to this approach: (1) it is relatively simple to implement, (2) it is random in nature, enabling the generation of an infinite number of different phantoms, and (3) it is computationally efficient, allowing generation of phantoms with many voids within reasonable time.

To summarize, the algorithm to generate foam phantoms works as follows:

(1) Create a list of *N*_p_ trial points, randomly placed within the main cylinder and satisfying the maximum height *z*_max_.

(2) Pick the trial point that results in the largest void (if multiple exist, randomly select one) and remove it from the list.

(3) Add a void at the picked trial point with the largest possible radius [equation (1)].

(4) Remove trial points that are inside the newly placed void from the list.

(5) Add new trial points to the list until the list has *N*_p_ points again, each randomly placed at a valid position (inside the main cylinder, outside any existing void, and satisfying the maximum height *z*_max_).

(6) Repeat steps (2) to (5) until *N* voids are placed.

Note that each foam phantom can be recreated deterministically given the following values: the number of voids *N*, the number of trial points *N*_p_, the maximum void size *r*_max_, the maximum height *z*_max_, and the random seed used for the random number generator.

There are several implementation tricks that can improve the computational performance of generating phantoms in practice. For example, the maximum possible radius [equation (1)] of each trial point can be precomputed and stored in a sorted data structure such as a skip list (Pugh, 1990 ▸) to enable fast access to the trial point with the largest possible radius. After placing a new void, the maximum radii have to be updated and reinserted in the sorted data structure, which can be efficiently done during step (4) above. For more details about these implementation tricks, we refer to the computer code that is available under an open-source license (Pelt, 2020 ▸).

### 2.3. Computing projections

The foam phantoms presented in this paper were developed for use in tomography research. As such, it is important that tomographic projections of these phantoms can be computed accurately and efficiently. Here, we assume that the projections are formed by the Radon transform: a measurement 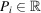 is computed by taking a line integral of the attenuation coefficients of the sample over the virtual X-ray *i*. The orientation and direction of the virtual ray depends on the tomographic acquisition geometry that is simulated. Measurements collected by 2D pixels with a certain area, which often represent real-world experiments better than individual rays, can be approximated by *supersampling*, *i.e.* averaging the measurements of multiple rays within a single pixel.

In many tomographic experiments, projections are formed by rotating the sample in front of a 2D detector (or, equivalently, rotating the detector around the sample) and acquiring separate 2D projection images at different angles. In these cases, the projection data are naturally described by a set of 2D projection images, each taken at a specific angle 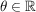 . Depending on the experimental setup, incoming rays of a single projection image are often assumed to be either parallel to each other (parallel-beam geometries) or to originate from a point source (cone-beam geometries).

In many existing comparisons between algorithms in which tomographic experiments are simulated, projections are formed by first discretizing the object on a discrete voxel grid and computing line integrals for the discrete object afterwards. As mentioned above, this approach can lead to the ‘inverse crime’, which can produce incorrect and misleading comparison results (Guerquin-Kern *et al.*, 2012 ▸). For the proposed foam phantoms, we prevent the inverse crime by computing projections analytically in the continuous domain, using the exact intersection between a ray and the phantom. Specifically, simulated X-ray projections of the proposed foam phantom are computed ray-by-ray. The line integral of the sample over a certain ray is computed by first computing the intersection of the main cylinder with that ray and subsequently subtracting the intersections with all voids, taking into account their attenuation factors. If we denote the intersection of ray *i* with the main cylinder by *L*_*i*_ and the intersection of ray *i* with void *j* by *l*(*i*, *j*), we can describe the projection *P*_*i*_ of ray *i* mathematically by

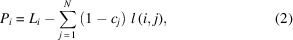

where *c*_*j*_ is the attenuation factor of void *j*, as described above. Note that for each void we have to both subtract the intersection that was counted in *L*_*i*_ and add the attenuation of the void itself, resulting in a factor of (1 − *c*_*j*_).

**Figure 3:**
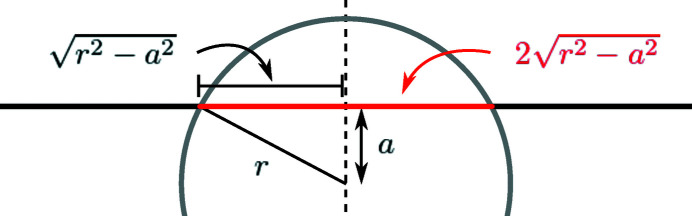
Schematic representation of the computation of the intersection (red) of a ray (black) and the main cylinder or a void (gray). The radius of the cylinder or void is given by *r*, and the closest distance between the ray and the center of the cylinder or void is given by *a*. The length of the intersection is then equal to 2(*r* ^2^ − *a* ^2^)^1/2^.

In parallel-beam geometries, the intersection *L*_*i*_ of the main cylinder with ray *i* can be computed by

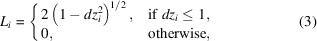

where *dz*_*i*_ is the shortest distance between any point along ray *i* and the *z*-axis (the rotation axis). The computation of this intersection is shown graphically in Fig. 3 ▸. For cone-beam geometries, it is also possible to analytically compute the intersection between a ray and the main cylinder, although it is more complicated than equation (3). For the sake of brevity, we refer to the computer code (Pelt, 2020 ▸) for more details about this computation.

The computation of intersections between rays and voids is similar to that of the main cylinder,

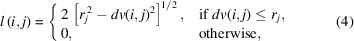

where *dv*(*i*, *j*) is the shortest distance between the center of void *j* and any point along ray *i*, and *r*_*j*_ is the radius of void *j*, as described above. The derivation of equation (4) is based on Fig. 3 ▸, extended to three dimensions. For more details about the analytic expression of the Radon transform of a sphere, we refer to Toft (1996 ▸). Note that shortest distances *dv*(*i*, *j*) can be computed efficiently by projecting the center *p*_*j*_ of void *j* along the direction of ray *i*. For parallel-beam geometries, all rays of a single projection image are parallel to each other, which enables precomputing the projections of all void centers for each projection image, significantly reducing the required computation time.

Detector noise can be simulated by applying Poisson noise to each measurement. First, a virtual photon count *I*_*i*_ for measurement *i* is computed using the Beer–Lambert law: 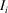 = 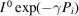 , where *I* ^0^ is the number of incoming photons and γ is a factor that controls the amount of radiation absorbed by the phantom. Afterwards, a noisy photon count 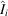 is computed by sampling from a Poisson distribution with *I*_*i*_ as the expected value. The noisy photon count is transformed back to a noisy measurement 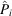 = 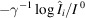 . In real-world tomographic experiments, other artifacts are often present in the measured data in addition to Poisson noise, for example due to source characteristics [*e.g.* beam hardening (Barrett & Keat, 2004 ▸)], additional photon interactions [*e.g.* free space propagation (Moosmann *et al.*, 2011 ▸)], optical effects (Ekman *et al.*, 2018 ▸), and detector defects (Miqueles *et al.*, 2014 ▸). Simulating such additional artifacts is not yet supported in the current version of the computer code. However, we note that it might be possible to include such artifacts in the future, either during the computation of projections within the code or as a post-processing step afterwards, possibly taking advantage of existing software packages that support them (Allison *et al.*, 2016 ▸; Faragó *et al.*, 2017 ▸).

### 2.4. 4D extensions

In recent years, improvements in radiation sources and detector equipment have increased interest in *dynamic* tomography of time-evolving samples (dos Santos Rolo *et al.*, 2014 ▸; Maire *et al.*, 2016 ▸; García-Moreno *et al.*, 2018 ▸). In these applications, samples are four-dimensional (4D) in nature, consisting of three spatial dimensions and one time dimension. To enable quantitative comparisons between algorithms for dynamic tomography (Kazantsev *et al.*, 2015 ▸; Mohan *et al.*, 2015 ▸; Van Nieuwenhove *et al.*, 2017 ▸; Nikitin *et al.*, 2019 ▸), 4D phantoms are needed. Similar to 3D phantoms, these phantoms should be challenging, representative, flexible, and suitable for data-driven applications. Here, we introduce such 4D phantoms by adapting the 3D foam phantoms described above, adding time-evolving aspects in different ways. Currently, the computer code includes three types of 4D extensions, which are described below. Additional 4D extensions are planned for future inclusion.

The first 4D extension is a *moving* phantom, in which the voids move along the *z*-axis. All voids move with the same velocity, but the velocity changes randomly during the experiment. The second extension is an *expanding* phantom, in which the size of all voids increases during the experiment. The third extension is an *infiltration* phantom, in which the voids are slowly filled by a material with a different attenuation coefficient than the initial void material. Specifically, all voids at a certain chosen height are filled at the start of the experiment. Then, each unfilled void with an edge close to a filled void is filled after a randomly chosen interval. This process is repeated until all voids are filled. Example phantoms for the three 4D extensions are shown in Fig. 6.

For all 4D extensions, several parameters can be chosen to adjust the time evolutions of the generated phantoms. After generating each 4D phantom, a dynamic tomography experiment can be simulated by virtually rotating the phantom during its time evolution, and computing projections as described in Section 2.3. Changes within the sample that happen *during* the acquisition of a single projection can be modeled by supersampling in time.

## 3. Experiments

### 3.1. Implementation details

Computer code to generate the proposed foam phantoms and simulate tomographic experiments is available as the open-source *foam_ct_phantom software* package (Pelt, 2020 ▸). The code is available for the Windows, MacOS, and Linux operating systems, and can be installed using the Conda package management system:[Chem scheme1] 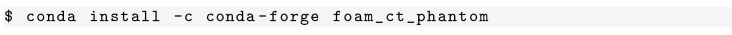 The code is implemented in the Python 3 (Van Rossum & Drake, 2009 ▸) programming language. Parts of the code with a high computational cost are implemented in the C programming language (Kernighan & Ritchie, 1988 ▸), using *OpenMP* (Dagum & Menon, 1998 ▸) for parallelization. Projections for cone-beam geometries can also be computed using NVidia Graphic Processor Units (NVidia, Santa Clara, CA, USA), which significantly reduces the required computation time. The GPU code is implemented using the *Numba* package (Lam *et al.*, 2015 ▸).

Generated phantoms and projection datasets are stored in HDF5 file containers (Folk *et al.*, 2011 ▸), using a simple custom data format that includes metadata about how the phantom or dataset was generated. A skip list (Pugh, 1990 ▸) is used to enable fast access to the trial point with the largest possible radius during generation of phantoms, and random numbers are generated using the Mersenne Twister algorithm (Matsumoto & Nishimura, 1998 ▸). The experiments in this paper were performed on a workstation with an AMD Ryzen 9 3900X CPU (AMD, Santa Clara, CA, USA), running the Fedora 32 Linux operating system. Experiments involving GPU computations were performed using a server with four NVidia GeForce RTX 2080 Ti GPUs (NVidia, Santa Clara, CA, USA), running the Fedora 30 Linux operating system.

### 3.2. Code examples

Below, we give a few code examples to show how the computer code can be used in practice to generate new phantoms, simulate tomographic experiments, and reconstruct projection data. First, new foam phantoms can be generated using the following Python code:[Chem scheme2] 
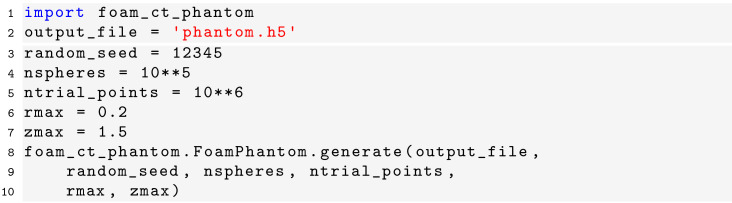


Here, the five parameters that determine the phantom shape (see Section 2.2) are given by 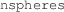 , 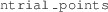 , 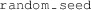 , 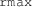 , and 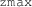 .

Once a phantom has been generated, parallel-beam projection data can be computed by the following Python code:[Chem scheme3] 
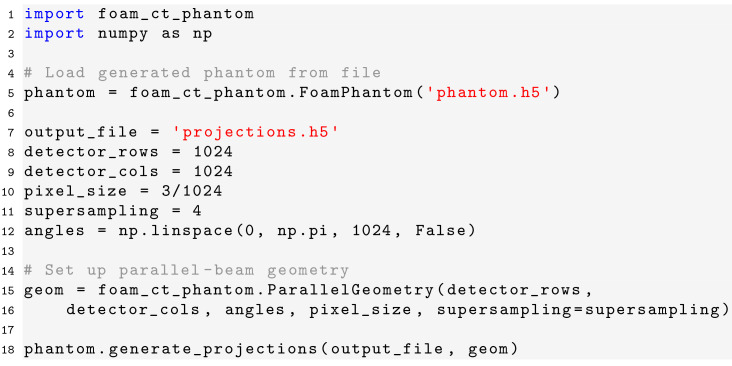


Here, the 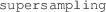 parameter controls the number of rays that are simulated within each pixel. Specifically, 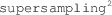 (*i.e.* 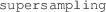 -squared) rays are simulated within each pixel, evenly distributed over the area of the pixel in a 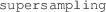 × 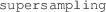 grid. The measured projection of a pixel is then the average value of the measurements of all rays within that pixel. For cone-beam projection data, only the geometry specification has to be changed to[Chem scheme4] 
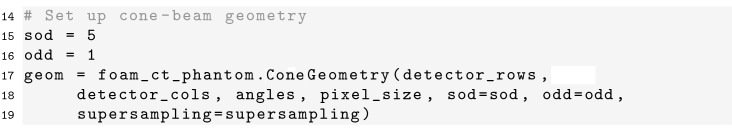


Here, 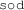 and 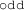 denote the source–object distance and object–detector distance, respectively.

The computer code also includes utility functions to assist in reconstructing the generated projection data using existing tomography toolboxes such as the *ASTRA* toolbox (Van Aarle *et al.*, 2016 ▸) and *TomoPy* (Gürsoy *et al.*, 2014 ▸). For the *ASTRA* toolbox, functions are included to convert defined geometries to equivalent ASTRA geometries:[Chem scheme5] 
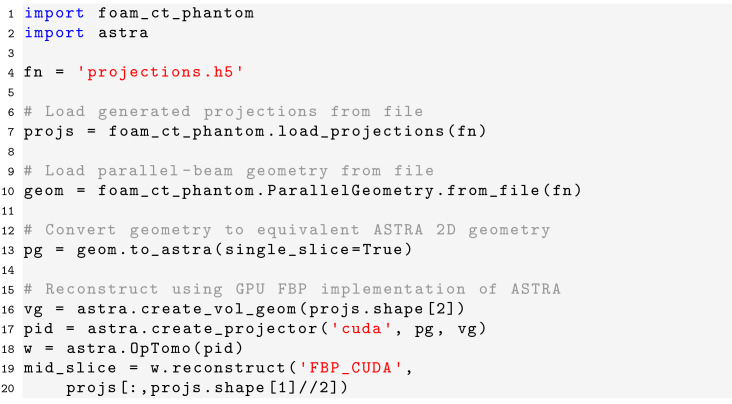


More code examples are included in the source code of the *foam_ct_phantom* package.

### 3.3. Phantom examples

**Figure 4:**
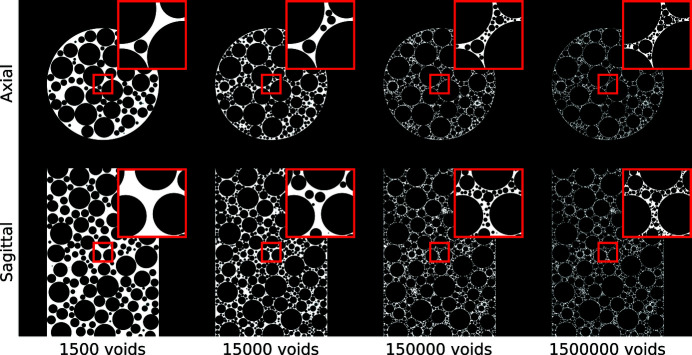
Generated foam phantoms with various numbers of voids. Given are the central axial slice and central sagittal (*i.e.* vertical) slice. A small region, indicated in red, is shown enlarged in the top right corner of each image.

In this section, we present several examples of generated phantoms, and investigate the effect of the various generation parameters on the phantom characteristics. As explained in Section 2.2, each phantom is defined by the number of voids *N*, the number of trial points *N*_p_, the maximum void size *r*_max_, and the maximum height *z*_max_. In the following, the values used for generating phantoms are *N* = 150000, *N*_p_ = 10^6^, *r*_max_ = 0.2, and *z*_max_ = 1.5 and all voids are filled with the background material, unless stated otherwise. Note that the code supports filling voids with other materials as well, making it possible to simulate objects with various characteristics, *e.g.* with low-contrast features. In Fig. 4 ▸, generated phantoms are shown for various numbers of included voids *N*. Since the other parameters are identical for all shown phantoms, the figure also shows how a phantom is generated by increasing the number of included voids. As expected, phantoms with a small number of voids mostly include relatively large voids, while the void size decreases with increasing numbers of included voids. In addition, the figures show both the large-scale and fine-scale features that are present phantoms with relatively large numbers of voids.

**Figure 5:**
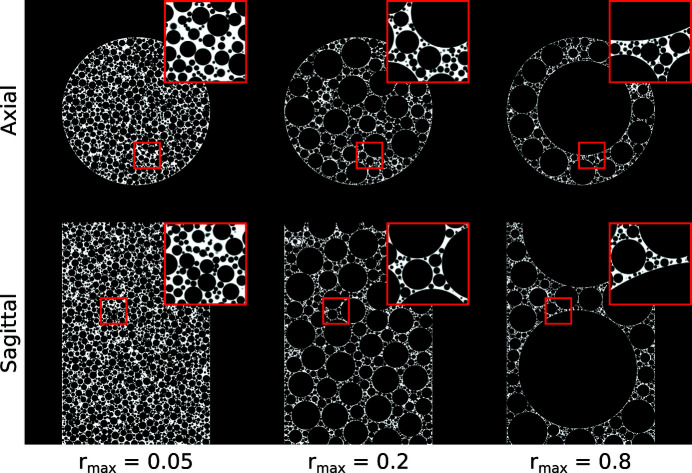
Generated foam phantoms with various values of the maximum possible void radius *r*_max_. Given are the central axial slice and central sagittal (*i.e.* vertical) slice. A small region, indicated in red, is shown enlarged in the top right corner of each image.

**Figure 6:**
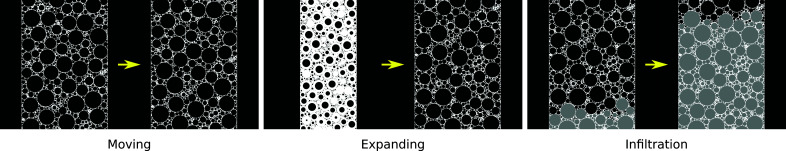
Examples of 4D extensions to the static 3D foam phantoms. In each case, an early time-point is shown on the left, and a later time-point is shown on the right. Given are an example of a moving phantom, in which the foam moves vertically, an expanding phantom, in which the voids grow in size, and an infiltration phantom, in which the voids fill with a different material over time.

In Fig. 5 ▸, generated phantoms are shown for three maximum void sizes *r*_max_ and *N* = 150000. The results show that the phantom features depend significantly on the choice of *r*_max_: for a relatively large maximum void size (*r*_max_ = 0.8), there are a few large voids present in the phantom and a large number of relatively small voids, as the large voids restrict the available space for the remaining voids. For a relatively small maximum void size (*r*_max_ = 0.05), most voids in the phantom have a similar size. The phantom with an intermediate maximum void size (*r*_max_ = 0.2) exhibits both characteristics to a lesser degree. These results show that the proposed phantom family can be used to simulate a wide variety of foam structures. Examples of the phantoms generated by the 4D extensions described in Section 2.4 are shown in Fig. 6 ▸.

### 3.4. Projection data and reconstruction examples

**Figure 7:**
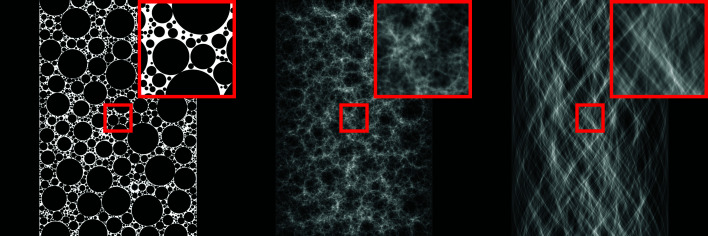
Example of the generation of parallel-beam projection data. Shown are the central sagittal (*i.e.* vertical) slice of a foam phantom (left), a parallel-beam projection of the phantom (middle), and a sinogram of the central axial (*i.e.* horizontal) slice (right). A small region, indicated in red, is shown enlarged in the top right corner of each image.

In this section, we present several examples of generated projection data and compare reconstruction results using several popular tomographic reconstruction algorithms. In all cases, we use the foam phantom generated with *N* = 150000, *N*_p_ = 10^6^, *r*_max_ = 0.2, and *z*_max_ = 1.5. Parallel-beam projections are computed for a detector with 2560 × 2160 pixels and 16 rays per pixel (*i.e.* 4 × 4 supersampling), mimicking a PCO.edge 5.5 sCMOS detector (PCO, Kelheim, Germany) that is commonly used at synchrotron tomography beamlines (Mittone *et al.*, 2017 ▸). The width and height of a detector pixel was set to 3/2560, resulting in a detector width of 3, with the sample (which has a fixed radius of 1) projecting on two-thirds of the detector width. Projections were computed for four imaging scenarios: ‘high-dose’, with a large number of noise-free projections; ‘noise’, with a large number of projections with a significant amount of Poisson noise applied; ‘few projections’, with a relatively low number of noise-free projections; and ‘limited range’, with a large number of noise-free projections acquired over less than 180°. Specific details about scenarios are given in Table 1 ▸, and example projection data are shown in Fig. 7 ▸.

We compare results for several popular tomographic reconstruction algorithms: the filtered backprojection method (FBP) (Kak *et al.*, 2002 ▸), SIRT (Kak *et al.*, 2002 ▸), CGLS (Scales, 1987 ▸), SART (Andersen & Kak, 1984 ▸), and SIRT and SART with additional nonnegativity constraints on the pixel values (Elfving *et al.*, 2012 ▸). All reconstruction images were computed using the optimized GPU implementations of the *ASTRA* toolbox (Van Aarle *et al.*, 2016 ▸). We compare the reconstructed images using three popular image quality metrics, the root mean square error (RMSE), peak signal-to-noise ratio (PSNR), and the multiscale structural similarity index (MS-SSIM) (Wang *et al.*, 2003 ▸). We also compare the images using two segmentation metrics, in which the images are segmented using thresholding, and Dice scores (Bertels *et al.*, 2019 ▸) are computed for voxels inside large voids (with radii *r*_*i*_ ≥ 0.1) and small voids (with radii *r*_*i*_ < 0.05). All metrics are computed with respect to a ground truth image that consists of a discretization of the foam phantom with the same number of voxels as the reconstructions and using 64 (4 × 4 × 4) sampling points per voxel. For the iterative algorithms, the number of iterations that minimizes the RMSE is used, which is determined using the Nelder–Mead method (Nelder & Mead, 1965 ▸) for CGLS and a simple grid search for all other algorithms.

**Figure 8:**
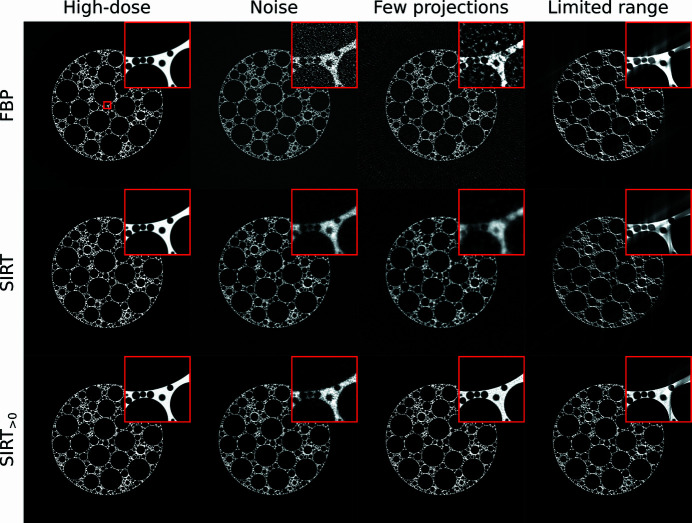
Reconstructed images of the central slice of a foam phantom, for various simulated experimental conditions (see Table 1 ▸). Given are results for FBP, SIRT, and SIRT with an additional nonnegativity constraint (SIRT_>0_).

In Table 2 ▸, the quality metrics are given for the central slice of the phantom and the four projection data scenarios given above. The results show that in most cases FBP produces images with the highest RMSE and lowest MS-SSIM values, the three unconstrained iterative methods (SIRT, CGLS, and SART) produce images with lower RMSE and higher MS-SSIM than FBP, and the iterative methods with nonnegativity constraints produce images with the lowest RMSE and highest MS-SSIM. However, the segmentation-based metrics show more nuanced results. For example, in the ‘limited range’ scenario, the Dice score for large voids of the FBP reconstruction is close to the Dice scores of the iterative algorithms, even though the RMSE is significantly higher and MS-SSIM significantly lower. This shows that, if the specific application of tomography would require only the analysis of large voids, the FBP algorithm would be sufficient, even though its image metrics are significantly worse than other methods. Similar results are shown in Fig. 8 ▸, in which a few selected reconstructed images are shown. Such results partly explain the continued popularity of FBP-like methods in practice (Pan *et al.*, 2009 ▸).

**Figure 9:**
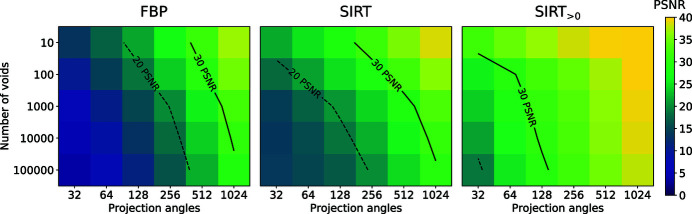
PSNR of reconstructed images of the central slice of a foam phantom as a function of the number of voids in the sample and the number of projection angles. Solid and dashed lines represent contour lines at 30 and 20 PSNR, respectively. Given are results for FBP, SIRT, and SIRT with an additional nonnegativity constraint (SIRT_>0_).

In Fig. 9 ▸, the PSNR of FBP, SIRT, and SIRT with a nonnegativity constraint is given as a function of both the number of projection angles and the number of voids in the phantom. In each case, data were generated with a low amount of Poisson noise (*I*_0_ = 10^7^) and a foam material that corresponds to an average absorption of 10% of virtual photons for a phantom with 150000 voids. The results show how the behavior of each reconstruction algorithm depends on the complexity of the scanned sample and the amount of acquired data. For example, the results show that the accuracy of FBP does not depend significantly on the complexity of the phantoms, while the accuracy of the SIRT algorithms is significantly improved for low-complexity samples compared with high-complexity samples. We note here that the proposed family of foam phantoms is especially well suited for performing such detailed comparisons, as the phantoms exhibit features at multiple scales, the level of complexity is tunable, and complete ground-truth information about the void positions and sizes is known. For another example of a detailed task-based analysis that uses the foam phantoms, we refer to Marchant *et al.* (2020 ▸).

### 3.5. Computation time

In this section, we present results for measurements of the required computation time for generating a foam phantom and computing projection data. A theoretical analysis of the required computation time for phantom generation is technically complicated due to the random nature of placing trial points. However, we hypothesize that, for large numbers of voids, the most time-consuming part is step (5) of the algorithm described in Section 2.2, and that the required computation time scales with 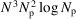 , where *N* is the number of voids and *N*_p_ the number of trial points used during generation. The various terms come from the fact that the required time for inserting an item in a skip list scales with 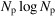 , the number of new trial points that have to be placed scales with *N*_p_, the number of voids that have to be checked for overlap for each new trial point scales with *N*, the number of required random trials until a valid position is found scales with *N* (since the available space decreases when more voids are placed), and step (5) has to be evaluated *N* times. The required computation time for simulating a projection scales with *N*_r_ *N*, where *N*_r_ is the number of simulated rays, which depends on the size of the detector and the amount of supersampling used.

**Figure 10:**
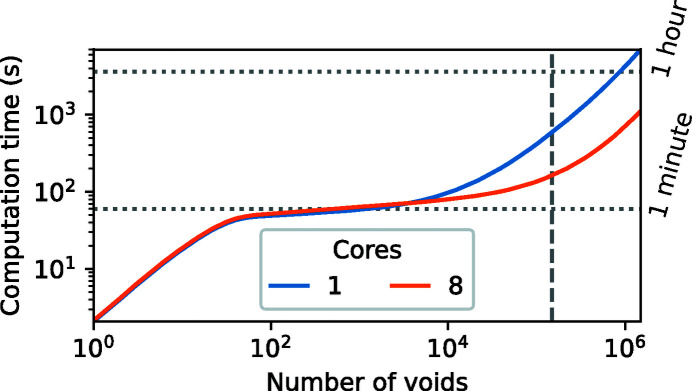
The required computation time for generating a foam phantom as a function of the number of voids in the phantom. The number of voids of the phantom that was used in most experiments in this paper (150000 voids) is indicated by the vertical dashed line.

In Fig. 10 ▸, the computation time required to generate a phantom with *r*_max_ = 0.2 is given as a function of the number of included voids. The results show that phantoms with a large number of voids, *e.g.* 100000 voids, can be generated within a few minutes. The results also show that using multiple CPU cores can significantly reduce the required computation time, especially for large numbers of voids. It is interesting to note that three different phases can be identified in Fig. 10 ▸. We hypothesize that different parts of the algorithm are dominant within each phase. During generation of the first 100 voids, we expect that most time is spent inserting newly placed trial points in the linked list data structure, which is not parallelized in the current implementation. Between 100 and around 10^5^ voids, we expect that most time is spent updating the maximum possible radius of each trial point, which is highly parallelizable. Finally, for more than 10^5^ voids, we expect that most time is spent finding valid positions while randomly placing new trial points, which is not parallelized in the current implementation as well. It may be possible to use such observations to reduce computation time for generating phantoms in the future.

**Figure 11:**
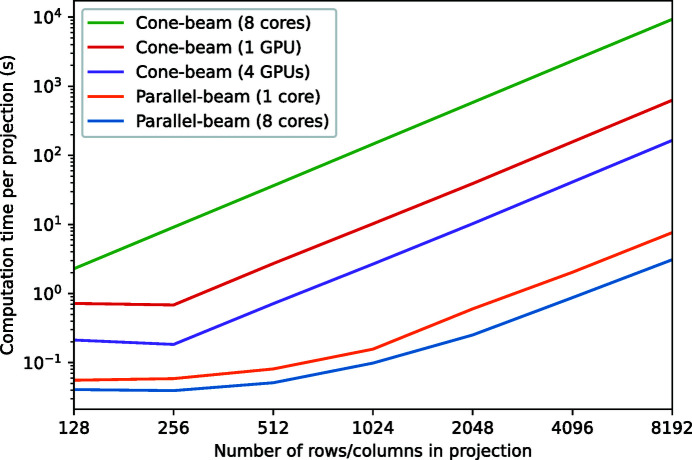
The required computation time for generating a tomographic projection for a phantom with 150000 voids as a function of the number of detector rows and columns. Results are given for using a single CPU core, eight CPU cores, a single GPU, and four GPUs.

In Fig. 11 ▸, the computation time for generating projections is given as a function of the number of rows and columns in each projection, for a phantom with 150000 voids and *r*_max_ = 0.2. The results show that it is possible to compute a parallel-beam projection with common high-resolution numbers of pixels, *e.g.* 1024 × 1024 pixels, in less that a tenth of a second using a modest multi-core CPU system. This computational efficiency makes it possible to generate full tomographic datasets within a few minutes. As explained in Section 2.3, computing cone-beam projections is more computationally demanding than computing parallel-beam projections. This is indeed visible in Fig. 11 ▸, which shows that even when using multiple CPU cores, generating a cone-beam projection can take considerable time. However, multiple GPUs can be used to significantly speed up these computations, reducing the required computation time to a few seconds per projection for common detector sizes.

## 4. Conclusion

In this paper, we introduced a family of foam-like phantoms for comparing the performance of tomography algorithms. The generated phantoms are challenging to reconstruct, representative of typical tomography experiments, and flexible, as projections can be calculated for various acquisition modes. In addition, an unlimited number of varying foam-like phantoms can be generated, enabling comparisons of data-driven algorithms. The phantoms consist of a main cylinder with a large number of randomly placed voids (*e.g.* more than 100000), resulting in foam-like structures with both large-scale and fine-scale features. We also introduced several 4D extensions to the static 3D phantoms, resulting in time-evolving phantoms for comparing algorithms for dynamic tomography.

Computationally efficient ways of both generating a phantom and simulating projection data for a given phantom were discussed, and a software package that implements these algorithms, the *foam_ct_phantom* package (Pelt, 2020 ▸), was introduced. Experimental results show that it is possible to generate phantoms on a modest workstation within a few minutes, and that projection data can be simulated for common high-resolution detector sizes within a few minutes as well. Comparisons between common reconstruction algorithms for several experimental settings show that it is possible to perform detailed analyses of algorithm performances using the proposed phantom family. These results show that the phantoms can be effectively used to make fair and informative comparisons between tomography algorithms.

## Figures and Tables

**Table 1:** Details of the projection datasets used for comparing reconstruction algorithms 50% absorption means that the γ parameter for noise generation was chosen such that the sample absorbed roughly 50% of the incoming photons.

	Geometry		Noise (Section 2.3)	
	Number of projections	Range	*I*_0_	Absorption
High-dose	1024	180°	N/A	N/A
Noise	1024	180°	250	50%
Few projections	128	180°	N/A	N/A
Limited range	682	120°	N/A	N/A

**Table 2:** Reconstruction results for various simulated experimental conditions (see Table 1 ▸). Additional nonnegativity constraints are indicated by ‘_>0_’. Metrics within 2% of the best metric in each column are shown in **bold**

		RMSE	MS-SSIM	Dice score			RMSE	MS-SSIM	Dice score	
		Full image	Full image	Large voids	Small voids		Full image	Full image	Large voids	Small voids
High-dose	FBP	0.035	0.901	**1.000**	**0.999**	Noise	0.394	0.335	0.938	0.922
	SIRT	0.034	0.940	**1.000**	**0.998**		0.115	0.668	**0.999**	**0.985**
	SIRT_>0_	**0.012**	**1.000**	**1.000**	**1.000**		**0.079**	**0.939**	**0.999**	**0.987**
	CGLS	0.033	0.936	**1.000**	**0.998**		0.111	0.692	**0.999**	0.974
	SART	0.033	0.939	**1.000**	**0.998**		0.178	0.397	**0.998**	0.962
	SART_>0_	**0.012**	**1.000**	**1.000**	**1.000**		0.095	0.883	**1.000**	**0.994**
Few projections	FBP	0.275	0.271	0.978	0.968	Limited range	0.174	0.741	**0.998**	0.973
	SIRT	0.141	0.547	**0.999**	0.961		0.135	0.774	**0.999**	**0.989**
	SIRT_>0_	**0.030**	**0.998**	**1.000**	**0.998**		**0.079**	**0.967**	**1.000**	**0.995**
	CGLS	0.139	0.545	**0.999**	0.962		0.134	0.769	**0.999**	**0.988**
	SART	0.139	0.545	**0.999**	0.962		0.135	0.775	**0.999**	**0.989**
	SART_>0_	**0.030**	**0.998**	**1.000**	**0.998**		**0.080**	**0.967**	**1.000**	**0.995**

## Footnotes

1 In rare cases, voids with the maximum size *r*_max_ can exist that touch neither the outside of the main cylinder nor another void. However, the radius of these voids cannot be increased as well, since their radius would become larger than the maximum allowed radius.
